# Cisplatin alters nitric oxide synthase levels in human ovarian cancer cells: involvement in p53 regulation and cisplatin resistance

**DOI:** 10.1038/sj.bjc.6604375

**Published:** 2008-05-27

**Authors:** E L Leung, M Fraser, R R Fiscus, B K Tsang

**Affiliations:** 1 Reproductive Biology Unit and Division of Gynecologic Oncology, Department of Obstetrics and Gynecology and Cellular and Molecular Medicine, University of Ottawa, Ottawa, Ontario, Canada K1Y 4E9; 2 Chronic Disease Program, Ottawa Health Research Institute, Ottawa, Ontario, Canada K1Y 4E9; 3 Department of Physiology, Faculty of Medicine and Centre of Gerontology and Geriatrics, Chinese University of Hong Kong, Shatin, NT, Hong Kong

**Keywords:** nitric oxide, ovarian cancer, apoptosis, NOS, p53, cisplatin

## Abstract

The present study determines if (1) basal protein levels of nitric oxide (NO) synthases (eNOS, iNOS, and nNOS) are different in cisplatin-sensitive (OV2008) and counterpart cisplatin-resistant (C13^*^) human ovarian cancer cells, (2) cisplatin alters NOS levels, (3) NO donor causes apoptosis and p53 upregulation, (4) NO donor sensitises C13^*^ cells to cisplatin via p53 upregulation (determined by p53 siRNA gene-knockdown), and (5) inhibition of endogenous NOS alters cisplatin-induced apoptosis. Basal iNOS levels were higher in OV2008 cells than in C13^*^ cells. Cisplatin upregulated iNOS, but dramatically reduced eNOS and nNOS, in OV2008 cells only. Failure of cisplatin to upregulate iNOS and downregulate eNOS/nNOS in cisplatin-resistant C13^*^ cells may be an aetiological factor in the development of resistance. The NO donor *S*-nitroso-*N*-acetylpenicillamine (SNAP) increased p53 protein levels and induced apoptosis in both cell types, and enhanced cisplatin-induced apoptosis in C13^*^ cells in a p53-dependent manner (i.e., enhancement blocked by p53 siRNA). Specific iNOS inhibitor 1400W partially blocked cisplatin-induced apoptosis in OV2008 cells. In cisplatin-resistant C13^*^ cells, blocking all NOSs with *N*^G^-amino-L-arginine dramatically changed these cells from cisplatin-resistant to cisplatin-sensitive, greatly potentiating cisplatin-induced apoptosis. The data suggest important roles for the three NOSs in regulating chemoresistance to cisplatin in ovarian cancer cells.

Cisplatin (CDDP) is a widely used first-line chemotherapeutic agent for ovarian cancer (Siddik, 2003), and the induction of apoptosis is a key determinant of CDDP sensitivity. Indeed, we and others have demonstrated that failure of CDDP to induce apoptosis is a major component of CDDP resistance (Sasaki *et al*, 2000; Yuan *et al*, 2003; Fraser *et al*, 2003a). CDDP induces DNA crosslinks, upregulation of p53, and subsequent p53-dependent apoptosis (Siddik, 2003). However, in CDDP-resistant ovarian cancer cells, failure to properly regulate p53 can contribute to the resistance (Fraser *et al*, 2003a, 2003b). Furthermore, basal activity of soluble guanylyl cyclase (sGC), an intracellular receptor for nitric oxide (NO), suppresses apoptosis in ovarian cancer cells, partly via inhibition of p53 accumulation and activation (Fraser *et al*, 2006). We hypothesised that basal sGC activity and regulation of p53 may depend on endogenous NO formed by one of the three NO synthases (NOSs), eNOS (also called NOS3), iNOS (also called NOS2), or nNOS (also called NOS1) and that resistance to CDDP in ovarian cancer cells may involve altered expression of the NOSs.

At high concentrations (typically >100 *μ*M), NO donors generate toxic concentrations of NO (e.g., >100 nM), inducing apoptosis in many mammalian cells (Fiscus, 2002; Fiscus *et al*, 2002), including cancer cells (Wink *et al*, 1998). However, NO at lower, more physiological levels (e.g., 1–20 nM) often has the opposite effect, preventing (or attenuating) the onset of apoptosis in many mammalian cells, including lung fibroblasts (Wink *et al*, 1993), human B lymphocytes (Mannick *et al*, 1994), and many normal and transformed neural cells (Fiscus, 2002; Fiscus *et al*, 2002). In most cells, the anti-apoptotic effects of low-level NO are attributed to the stimulation of sGC with subsequent elevation of cyclic guanosine monophosphate (cGMP) levels and increased activation of protein kinase G (PKG). The anti-apoptotic role of cGMP/PKG signalling pathway has been further established using natriuretic peptides, atrial natriuretic peptide, and B-type/brain natriuretic peptide (BNP), which activate cGMP/PKG via an alternate pathway (i.e., activation of particulate guanylyl cyclase). Atrial natriuretic peptide and BNP are exceptionally effective inhibitors of stress-induced apoptosis in PC-12 phaeochromocytoma and in NG108-15 neuroblastoma–glioma hybrid cells, including suppressing the toxic/pro-apoptotic effects of high-level NO (Fiscus *et al*, 2001; Cheng Chew *et al*, 2003). Even basal levels of cGMP, generated primarily by basal sGC activity, and subsequent partial activation of PKG are sufficient (in fact, necessary) to prevent spontaneous apoptosis in certain cells, including NG108-15 cells (Fiscus, 2002; Fiscus *et al*, 2002), immortalised uterine epithelial cells (Chan and Fiscus, 2003), and human ovarian cancer cells (Fraser *et al*, 2006). Because sGC is activated by NO at very low concentrations (i.e., 50% of maximal sGC activity stimulated by only 4 nM NO; Bellamy *et al*, 2002), low endogenous level of NO, generated by one of the three NOSs, may be responsible for stimulating basal sGC activity and inhibiting apoptosis in these cells.

Previously, melanoma cells (Tang and Grimm, 2004), transformed *β*-cell line RINm5F (Tejedo *et al*, 2001), and transformed human leukocytes (Mannick *et al*, 1997) were reported to generate endogenous NO capable of suppressing apoptosis. Endogenous NO is also believed to be responsible for aggressive tumour progression, metastasis, and poor survival in cancer patients with tumours exhibiting increased iNOS expression (Thomsen and Miles, 1998). However, other studies focusing on ovarian cancer have shown that overexpression of iNOS correlates with increased apoptosis (Son and Hall, 2000; Rieder *et al*, 2001). Thus, endogenous NO may contribute to either anti-apoptotic or pro-apoptotic effects in cancer cells, most likely depending on cell type, local NO levels, source of NO production, and other conditions (e.g., concurrent overproduction of superoxide anion, which can combine with NO to form the toxic pro-oxidant peroxynitrite) (Fiscus, 2002; Fiscus *et al*, 2002).

The present study determines whether eNOS, iNOS, and nNOS are involved in regulating p53 accumulation and CDDP resistance in ovarian cancer cells. CDDP-sensitive OV2008 cells and its resistant variant C13^*^ cells were used. Effects of NO donor and NOS inhibitors on CDDP-induced p53 regulation and apoptosis were also studied. Overall, the present study shows that all three isoforms of NOS are expressed in both cell lines, but protein levels are differentially regulated by CDDP in CDDP-sensitive and CDDP-resistant cells. Furthermore, the data suggest that iNOS contributes to CDDP-induced apoptosis in CDDP-sensitive cells, whereas eNOS/nNOS contributes (in a p53-independent manner) to chemoresistance by suppressing CDDP-induced apoptosis in isogenic CDDP-resistant cells.

## MATERIALS AND METHODS

### Reagents

Chemicals were purchased from the following suppliers: Hoechst 33258, phenylmethlysulphonyl fluoride (PMSF), sodium orthovanadate (Na_3_V0_4_), and aprotinin (Sigma, St Louis, MO, USA); *S*-nitroso-*N*-acetylpenicillamine (SNAP) and rabbit monoclonal anti-iNOS, anti-eNOS, and anti-nNOS antibodies (Calbiochem-Novabiochem, La Jolla, CA, USA); mouse monoclonal anti-p53, clone DO-1 (Santa Cruz Biotechnologies, Santa Cruz, CA, USA); mouse monoclonal anti-GAPDH antibody (ab8245) (Abcam, Cambridge, UK); p53-siRNA (Cell Signaling Technology, Beverly, MA, USA); pre-stained SDS-PAGE standards (Bio-Rad, Hercules, CA, USA); scrambled control siRNA (Dharmacon, Lafayette, CO, USA); specific iNOS inhibitor 1400W and general NOS inhibitor *N*^G^-amino-L-arginine (L-NAA) (Alexis Biochemicals, San Diego, CA, USA).

### Cell lines and culture

Two counterpart human epithelial ovarian cancer cell lines, OV2008 and C13^*^ cells, were used. Cisplatin-sensitive OV2008 cells was derived from ovarian cystadenocarcinoma patient without prior chemotherapy, and its CDDP-resistant variant C13^*^ was established after *in vitro* CDDP challenge. Wild-type TP53 status of these cells has been previously demonstrated by direct sequencing (Fraser *et al*, 2008). Cells were cultured as described previously (Fraser *et al*, 2006).

### Hoechst 33258 staining

Cells were handled and stained with Hoechst 33258 as previously described (Fraser *et al*, 2006). Counting was carried out blinded to sample identity, to reduce experimental bias.

### Protein extraction and western blotting

Cells were pelleted and lysed in ice-cold lysis buffer (pH 7.4) containing 50 mM Hepes, 150 mM NaCl, 1.5 mM MgCl_2_, 1 mM EGTA, 100 mM NaF, 10 mM NaPPi, 10% glycerol, and 1% Triton X-100. Protease inhibitors PMSF (1 mM), aprotinin (125 000 IU ml^−1^), and Na_3_VO_4_ (1 mM) were added to the lysis buffer freshly. Cell lysates were sonicated briefly for three cycles (5 s per cycle, 0 °C). Sonicates were incubated on ice for 1 h and pelleted by centrifugation (15 000 **g**; 20 min). Protein concentration was determined using Bio-Rad DC Protein Assay Kits. Equal amounts of proteins (50–100 *μ*g) were loaded and resolved by 15% SDS-PAGE and electrotransferred (110 V, 1 h) onto nitrocellulose membranes (Bio-Rad) as previously described (Fraser *et al*, 2006). Membranes were blocked (at room temperature, 1 h) with 5% Blotto (Tris-HCl (10 mM; pH 8.0), NaCl (150 mM), Tween 20 (0.05%, v/v; TBS-Tween 20) containing skim milk (5%; w/v)), then incubated overnight with primary antibodies iNOS, eNOS, nNOS, and p53 (1 : 1000) or GAPDH (1 : 200 000)), and subsequently with the appropriate horseradish peroxidase-conjugated secondary antibody (1 : 2000 in 5% Blotto; at room temperature for 1 h). The membranes were washed with TBS-Tween 20 thrice (15 min per wash), peroxidase activity was visualised with ECL reagent (Amersham Biosciences), and exposed to Hyperfilm ECL (Amersham Biosciences, Buckinghamshire, UK). Signal intensity was determined densitometrically by Scion Image Software (version 4.02; Scion Corporation, Frederick, MD, USA).

### RNA interference

After plating cells for 18 h, 6 *μ*l of RiboJuice (Novagen, San Diego, CA, USA) was added to 244 *μ*l of RPMI medium without serum. The mixture was vortexed and incubated for 5 min at room temperature. After incubation, p53 siRNA (Cell Signaling Technology) and control non-targeting siRNA (Dharmacon) at final concentration of 50 nM were mixed with the transfection reagents. The mixture was incubated at room temperature for 15 min. During this period, the culture media were removed from the cells and the cells were washed once with PBS. The siRNA mixture was added in drop by drop to each well to ensure maximal coverage, and an additional 1250 *μ*l of complete media was added to bring the total volume to 1.5 ml. The cells were incubated for 6 h and then the media were removed and replaced with fresh, complete media for 24 h. Downregulation was confirmed by western blot analysis.

### Statistical analysis

Results are expressed as mean±s.e.m. of four or five independent experiments. Statistical analysis was performed by one-way or two-way ANOVA using PRISM software (Version 3.0; GraphPad, San Diego, CA, USA). Bartlett's tests were used to establish homogeneity of variance on the basis of differences among s.d. Differences between experimental groups were determined by Bonferroni multiple comparison test. *P*<0.05 was considered to be significant.

## RESULTS

### Basal iNOS, eNOS, and nNOS levels are different in CDDP-sensitive (OV2008) cells than in CDDP-resistant (C13^*^) cells and are differentially regulated by CDDP *in vitro*

**Figure 1:**
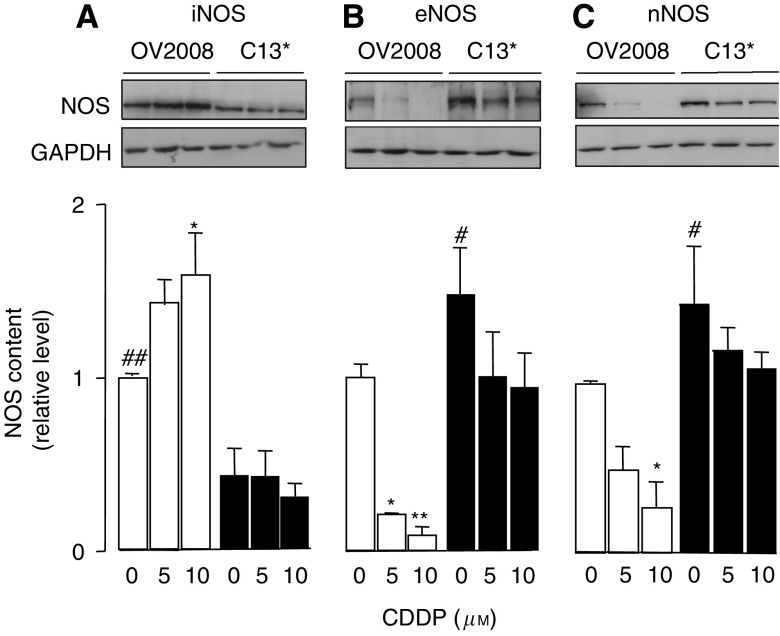
Protein levels of iNOS, eNOS, and nNOS in cisplatin-sensitive OV2008 and in cisplatin-resistant C13^*^ cells were determined using western blot analysis. (**A**) Basal iNOS levels were significantly higher in OV2008 cells compared with C13^*^ cells. Cisplatin (CDDP, 10 *μ*M, 24 h) significantly upregulated iNOS in OV2008, but not in C13^*^ cells. (**B** and **C**) Basal levels of eNOS and nNOS were significantly lower in OV2008 cells compared with C13^*^ cells. Cisplatin (5 and 10 *μ*M, 24 h) significantly downregulated both eNOS and nNOS in OV2008, but not in C13^*^, cells. The bar graphs show mean±s.e.m. of protein levels of five independent experiments. ^*^P<0.05, compared with control (no cisplatin). ^**^P<0.01, compared with control (no cisplatin). ^#^*P*<0.05, comparing basal levels in control OV2008 and in C13^*^ cells. ^##^*P*<0.01, comparing basal levels in control OV2008 and in C13^*^ cells.

To assess the protein levels of basal and CDDP-induced NOS in chemosensitive and chemoresistance cells, we cultured OV2008 ovarian cancer cells and the isogenic chemoresistant variant C13^*^ in the absence or presence of CDDP (0–10 *μ*M, 24 h; DMSO control) and evaluated iNOS, eNOS, and nNOS contents by western blot. As shown in Figure 1A, basal iNOS levels in OV2008 cells are significantly higher than those in C13^*^ cells (*P*<0.05) and are significantly upregulated by CDDP in the chemosensitive cells, but not in the resistant variant cells (*P*<0.01). In contrast, basal eNOS and nNOS levels in CDDP-sensitive OV2008 cells were significantly (*P*<0.05) lower than in CDDP-resistant C13^*^ cells (Figure 1B and C) and were significantly downregulated after CDDP treatment (*P*<0.01). As observed with iNOS, CDDP failed to significantly alter eNOS and nNOS content in C13^*^ cells, although a slight downwards trend was observed. The data are consistent with the hypothesis that iNOS, eNOS, and nNOS are differentially regulated in chemosensitive (OV2008) and chemoresistant (C13) cells and that CDDP resistance is associated with low iNOS content and high levels of eNOS and nNOS in C13 cells, which are unresponsive to CDDP.

### Effects of a NO donor on p53 and CDDP sensitivity

**Figure 2:**
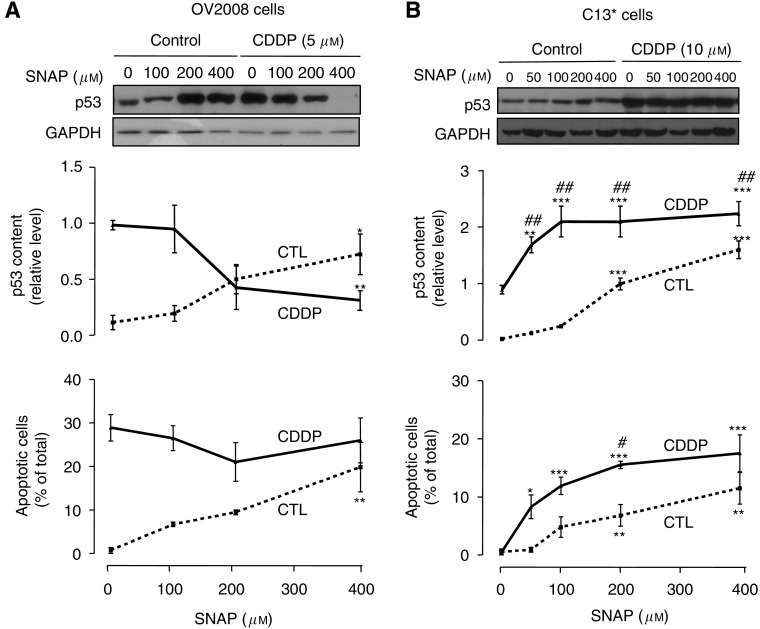
(**A**) OV2008 cells were cultured with SNAP (0, 100, 200, 400 *μ*M) for 24 h, followed by 5 *μ*M cisplatin (CDDP) for another 24 h. p53 protein content was assessed by western blot analysis. Results were normalised for protein loading by re-probing with anti-GAPDH antibody. Western blots are representative of four independent experiments. Graphs show mean±s.e.m. of protein contents of four independent experiments. *S*-nitroso-*N*-acetylpenicillamine alone (400 *μ*M, ^*^P<0.05) increased basal p53 content, but decreased cisplatin-induced upregulation of p53 (^**^P<0.01) in chemosensitive OV2008 cells. *S*-nitroso-*N*-acetylpenicillamine (400 *μ*M) alone significantly (^**^P<0.01) increased apoptosis. However, when used in combination with cisplatin, SNAP did not further enhance cisplatin-induced apoptosis. (**B**) C13^*^ cells were cultured with SNAP (0, 50, 100, 200, 400 *μ*M) for 24 h, followed by 10 *μ*M cisplatin for another 24 h. Western blots are representative of five independent experiments. Graphs show mean±s.e.m. of p53 protein content and apoptosis of five independent experiments. *S*-nitroso-*N*-acetylpenicillamine (200 and 400 *μ*M) alone significantly (^***^P<0.001) upregulated p53 levels. With cisplatin co-treatment, SNAP at 50, 100, 200, and 400 *μ*M significantly (^**^P<0.01, ^***^P<0.001) increased p53 levels above control. p53 upregulation induced by SNAP plus cisplatin was significantly (^##^*P*<0.01) larger than with SNAP alone. *S*-nitroso-*N*-acetylpenicillamine (200 and 400 *μ*M) alone significantly (^**^P<0.01) induced apoptosis and sensitised chemoresistant C13^*^ cells to cisplatin-induced apoptosis (SNAP at 200 *μ*M, ^#^*P*<0.05).

Because we observed differential regulation of NOS isoforms in chemosensitive and chemoresistant ovarian cancer cells, we next asked whether NO itself could influence sensitivity to CDDP-induced apoptosis. Moreover, as p53 is a key determinant of CDDP sensitivity (Fraser *et al*, 2003b) and is regulated by the cGMP pathway (Fraser *et al*, 2006), we also examined the effects of the NO donor SNAP (Bellamy *et al*, 2002; Fiscus *et al*, 2002) on basal and CDDP-induced p53 contents. OV2008 or C13^*^ cells were pretreated with SNAP for 24 h, followed by treatment with CDDP for a further 24 h. As shown in Figure 2A, SNAP significantly upregulated p53 content (*P*<0.05) and induced apoptosis (*P*<0.01) in OV2008 cells. Interestingly, although CDDP alone also upregulated p53 and induced apoptosis in these cells, co-treatment with SNAP and CDDP resulted in a decrease in p53 content and no further increase in CDDP-induced apoptosis. Similarly, in C13^*^ cells, SNAP alone upregulated basal p53 and induced apoptosis in C13^*^ cells. However, in contrast to the chemosensitive cells, pretreatment with SNAP facilitated the CDDP-induced upregulation of p53 (*P*<0.01) and sensitised the cells to CDDP-induced apoptosis (*P*<0.05) (Figure 2B). These data suggest that high-level NO, released from an NO donor such as SNAP (Bellamy *et al*, 2002; Fiscus *et al*, 2002), upregulates p53 and induces apoptosis in resistant ovarian cancer cells and sensitises these cells to pro-apoptotic effects of CDDP.

### p53 siRNA reduces NO donor-induced apoptosis in both OV2008 and C13^*^ cells and sensitises C13^*^ cells to CDDP

**Figure 3:**
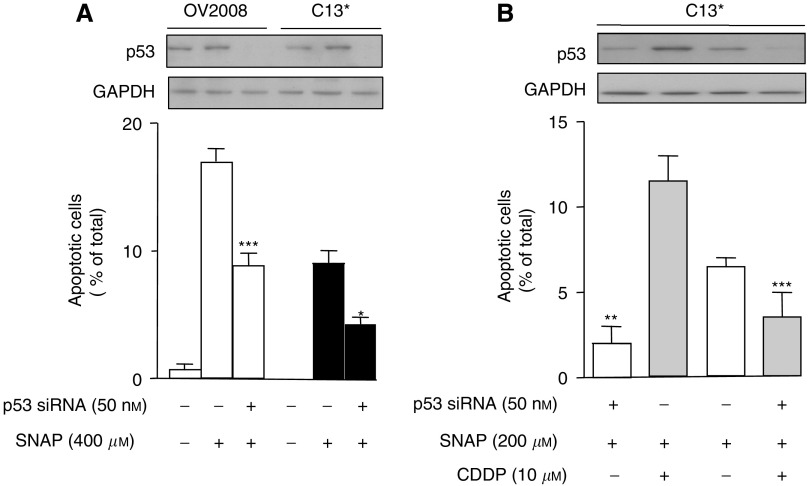
(**A**) Both OV2008 and C13^*^ cells were transfected with p53-specific siRNA (50 nM; scrambled sequence as control) for 24 h, and subsequently treated with 400 *μ*M SNAP for another 24 h. Western blots are representative of four independent experiments. Graphs show mean±s.e.m. of four independent experiments. Western blots show that p53 was successfully knocked down. ^*^P<0.05, ^***^P<0.001, compared with SNAP treatment alone. (**B**) C13^*^ cells were transfected with p53-specific siRNA (50 nM; scrambled sequence as control) for 24 h, and subsequently treated with 200 *μ*M SNAP before cisplatin (CDDP, 10 *μ*M) treatments for another 24 h. Western blots are representative of four independent experiments and graphs show mean±s.e.m. from four independent experiments. ^**^P<0.01, compared with SNAP alone, ^***^P<0.001, compared with SNAP plus cisplatin (without siRNA).

We and others have demonstrated that p53 is an important determinant of sensitivity to CDDP-induced apoptosis in ovarian cancer cells. To test the role of p53 in the chemosensitising effects of high concentrations of NO, we transfected chemosensitive and chemoresistant cells with p53 siRNA and assessed the effects of p53 downregulation on SNAP-mediated sensitisation to CDDP-induced apoptosis. As shown in Figure 3A, p53 levels were successfully silenced by p53 siRNA, as compared with control siRNA transfection in both OV2008 and C13^*^ cells. Importantly, the downregulation of p53 significantly attenuated SNAP-induced apoptosis in both OV2008 and C13^*^ cells (*P*<0.001 and *P*<0.01, respectively), suggesting that high concentrations of NO induce apoptosis in a p53-dependent manner.

To evaluate whether p53 is implicated in the sensitisation of cells to CDDP-induced apoptosis by high concentrations of NO, we transfected C13^*^ cells with p53 siRNA (or control) followed by treatment with SNAP and/or CDDP. As shown in Figure 3B and consistent with the results shown in Figure 2B, SNAP sensitised C13^*^ cells to CDDP-induced apoptosis, and this was significantly suppressed by the downregulation of p53 (*P*<0.001), suggesting that p53 is necessary for NO donor-induced sensitisation to CDDP.

### iNOS is required for CDDP-induced p53 accumulation and apoptosis

**Figure 4:**
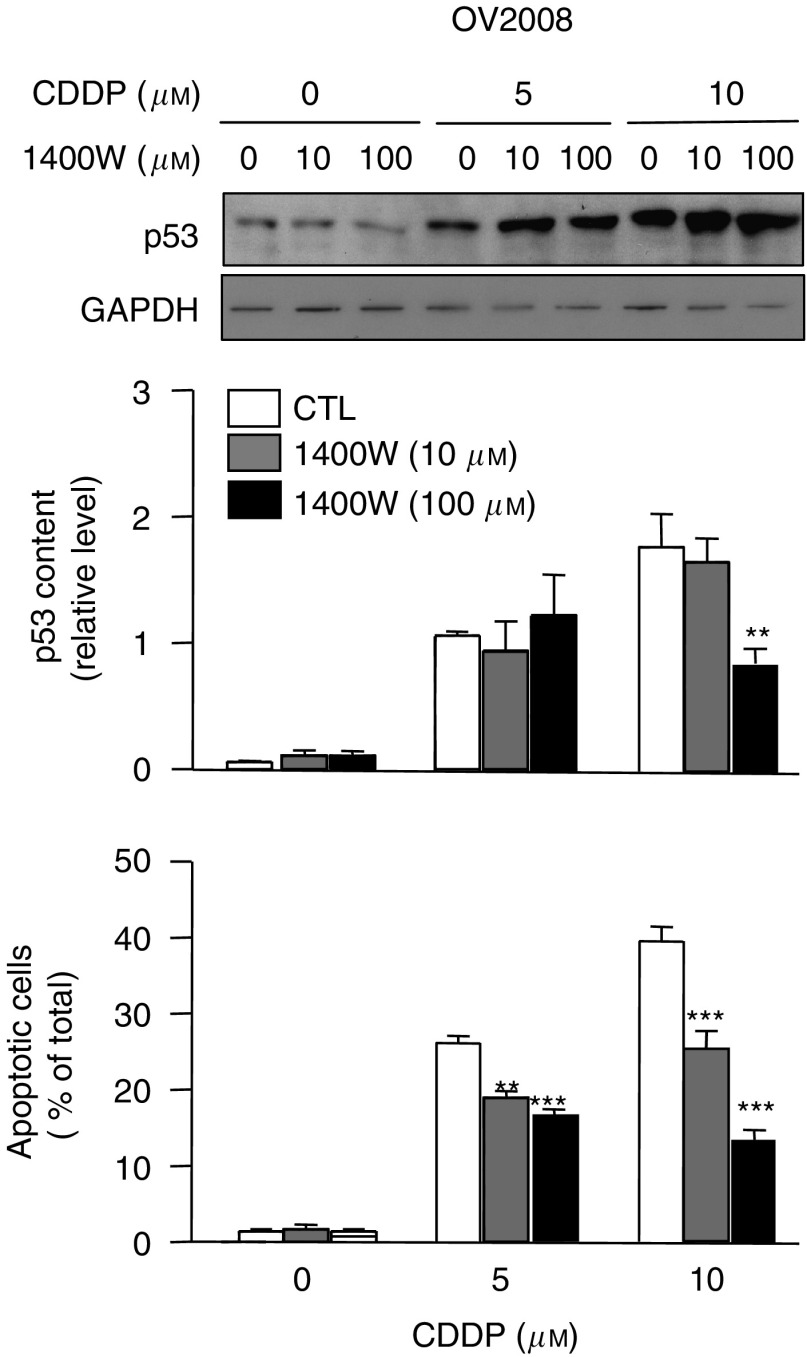
OV2008 cells were cultured with 1400W (0, 10, 100 *μ*M) for 24 h and further treated with cisplatin (CDDP; 0, 5, 10 *μ*M) for another 24 h. Protein content of p53 was assessed by western blot analysis and apoptosis by Hoechst staining. Western blots are representative of four independent experiments. Graphs show mean±s.e.m. of four independent experiments. ^**^P<0.01, ^***^P<0.001, compared with control.

We sought to assess whether iNOS is required for the pro-apoptotic effects of CDDP. To that end, we treated OV2008 cells with the specific iNOS inhibitor 1400W (Thomsen *et al*, 1997) (100 *μ*M, 24 h) and evaluated CDDP-induced p53 content and apoptosis. As shown in Figure 4, 1400W partially blocked CDDP-induced p53 accumulation. However, this effect was observed only with higher concentrations of 1400W. In contrast, the pro-apoptotic effects of CDDP were significantly reduced by both higher and lower (10 *μ*M) concentrations of 1400W, suggesting that CDDP-induced apoptosis is dependent on endogenous iNOS activity.

### Inhibition of all endogenous NOSs does not alter CDDP-induced p53 content in CDDP-resistant C13^*^ cells, but sensitises them to CDDP

Because CDDP-resistant C13^*^ cells show higher basal protein levels of eNOS and nNOS and are resistant to CDDP-induced downregulation of eNOS and nNOS, we hypothesised that eNOS/nNOS activity may play an important role in the resistance to CDDP in C13^*^ cells. To test this hypothesis, L-NAA, a general NOS inhibitor that inhibits all three isoforms of NOS, was used (Reif and McCreedy, 1995). Unlike 1400W, which is highly specific for iNOS, none of the commercially available inhibitors of eNOS or nNOS are highly specific. Thus, to effectively inhibit eNOS and nNOS in CDDP-resistant C13^*^ cells, we used the multitargeted NOS inhibitor L-NAA.

**Figure 5:**
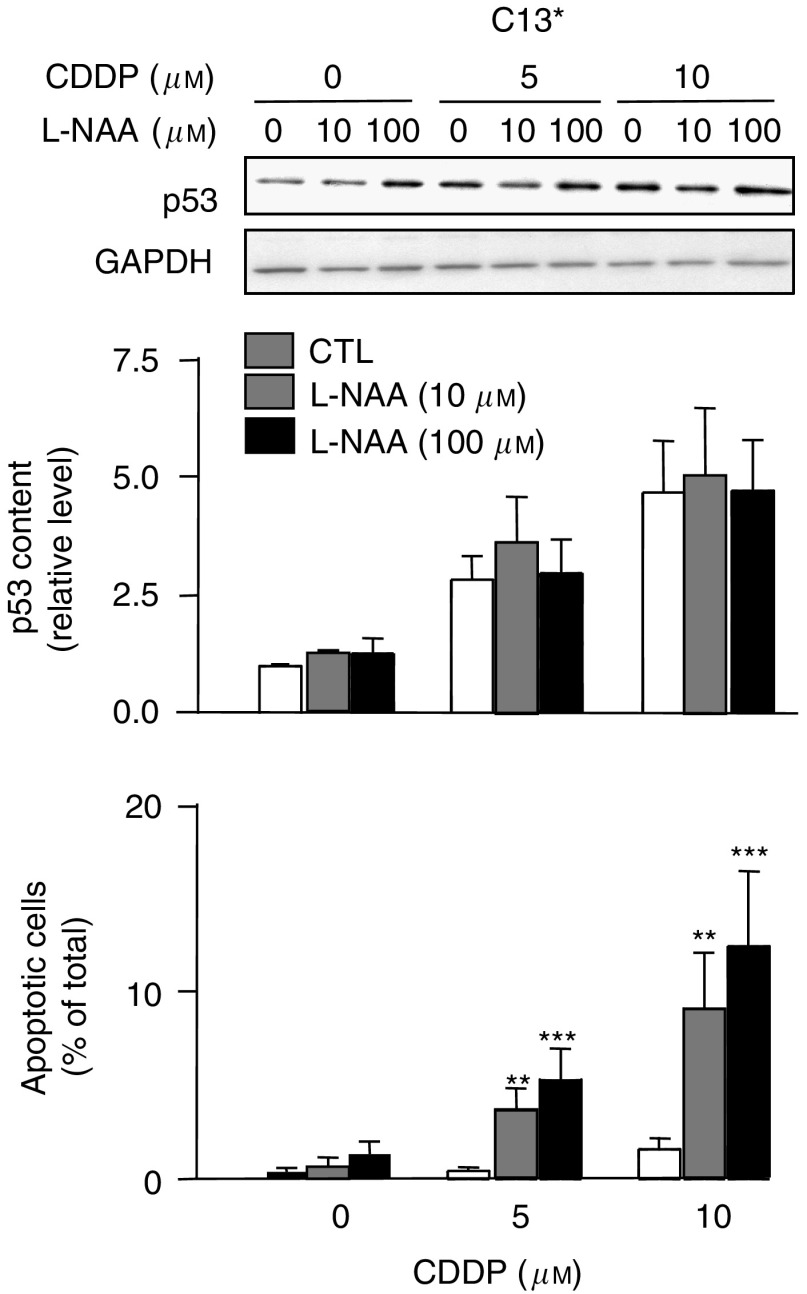
Inhibition of all NOSs using *N*^G^-amino-L-arginine (L-NAA; 0, 10, 100 *μ*M) for 24 h, followed by cisplatin (0, 5, 10 *μ*M) for additional 24 h. Western blots are representative of four independent experiments and graphs shows mean±s.e.m. from four independent experiments. ^**^P<0.01, ^***^P<0.001.

Figure 5 shows western blot analysis of C13^*^ cells pretreated with L-NAA (0, 10, or 100 *μ*M) for 24 h, followed by CDDP (0, 5, or 10 *μ*M) for a further 24 h. There was no significant effect of L-NAA on p53 levels in any of the CDDP-treated groups, suggesting that neither eNOS nor nNOS affect p53 content. In contrast, L-NAA (both 10 and 100 *μ*M) dramatically enhanced the pro-apoptotic effects of CDDP. These data suggest that low-level NO generated by eNOS and nNOS contributes to the suppression of CDDP-induced apoptosis in C13^*^ cells.

## DISCUSSION

The present study demonstrates that protein levels of the different NOS isoforms or co-treatment with the NO donor SNAP regulates induction of apoptosis and sensitisation to CDDP in human ovarian cancer cells. CDDP upregulates iNOS protein levels and markedly downregulates eNOS and nNOS protein levels in CDDP-sensitive OV2008 cells but has no significant effect on the levels of the three NOSs in CDDP-resistant C13^*^ cells, suggesting that differential regulation of the NOSs may contribute to CDDP resistance. The data using NOS inhibitors suggest that iNOS contributes to CDDP-induced apoptosis in OV2008 cells, whereas eNOS/nNOS contributes to CDDP-resistant in C13^*^ cells.

Endogenous NO is produced in mammalian cells by the three NOSs, namely eNOS (NOS3), originally found constitutively expressed in endothelial cells; iNOS (NOS2), first found to be induced by cytokines in activated macrophages; and nNOS (NOS1), originally discovered to be constitutively expressed in neural cells (Forstermann *et al*, 1995; Fiscus, 2002; Fiscus *et al*, 2002). All of these NOS isozymes have now been found in many other cell types. Typically, eNOS and nNOS produce NO at low or physiological levels, which play important roles in the normal regulation of the cardiovascular and neural systems (Fiscus, 2002; Fiscus *et al*, 2002). Low-level NO (e.g., 1–20 nM) binds to the heme moiety of sGC, increasing its enzymatic activity, which in turn results in the elevation of cGMP levels and increased activity of PKG in mammalian cells. Stimulation of the sGC/cGMP/PKG pathway by low-level NO contributes to cytoprotective/anti-apoptotic effects in many types of mammalian cells (Fiscus, 2002; Fiscus *et al*, 2002). In contrast, iNOS typically produces toxicity-associated NO. The toxicity may result from NO combining with superoxide anion, forming peroxynitrite, a powerful oxidising substance that induces oxidative and nitrosative stress, and apoptosis in mammalian cells (Fiscus, 2002; Fiscus *et al*, 2002). The present study shows that all three isoforms of NOS are expressed in human ovarian cancer cells and that the NOSs are differentially expressed in CDDP-sensitive and CDDP-resistant cells, suggesting potential involvement in chemoresistance.

Previous reports have demonstrated that pro-inflammatory cytokines induced iNOS expression and apoptosis in ovarian cancer cells (Son and Hall, 2000; Rieder *et al*, 2001). The current study extends these findings by demonstrating that the pro-apoptotic effects of iNOS may be mediated, in part, through a p53-dependent pathway. This finding may have significant implications with regard to the molecular mechanisms of chemosensitivity in ovarian cancer cells, as p53 plays a key role in this process (Fraser *et al*, 2003b). This hypothesis is further supported by the observations that in normal human fibroblasts and liver epithelial cells, iNOS induction stimulates p53 accumulation and apoptosis (Forrester *et al*, 1996). Many tumour cells express higher levels of iNOS and it is believed that NO generated by iNOS contributes to p53 upregulation and induction of apoptosis in cancer cells (Ambs *et al*, 1997). However, to date, reports showing direct functional evidence linking NO, p53, and chemosensitivity in ovarian cancer cells have been lacking. Thus, the present study represents the first report directly demonstrating the role of p53 in high-level NO-induced apoptosis (using siRNA gene knockdown) and the role of the three endogenous NOSs in regulating sensitivity to CDDP in ovarian cancer cells.

The present study shows that the NO donor SNAP, when used at higher concentrations (200 and 400 *μ*M) that generate toxic levels of NO, induces apoptosis in both CDDP-sensitive (OV2008) and CDDP-resistant (C13^*^) ovarian cancer cells. Furthermore, SNAP (50–400 *μ*M) caused concentration-dependent enhancement of CDDP-induced p53 upregulation in C13^*^ cells, sensitising these cells to pro-apoptotic effects of CDDP. Gene knockdown with p53 siRNA prevented this sensitisation, demonstrating that high concentrations of NO contribute to CDDP sensitivity through a p53-dependent mechanism.

Although depletion of endogenous NO in melanoma cells by NO scavengers enhanced CDDP-induced apoptosis and cell cycle arrest in a p53-dependent manner (Tang and Grimm, 2004), a direct connection between p53 and CDDP-induced apoptosis was not established. The present study shows that CDDP sensitisation induced by high levels of NO donor in ovarian cancer cells directly involves p53 upregulation, illustrated by our p53 siRNA gene knockdown experiments (Figure 3). In contrast, CDDP sensitisation induced by inhibition of all three forms of NOS in C13^*^ ovarian cancer cells (Figure 5) did not change p53 levels in ovarian cancer cells. Thus, the role of p53 in CDDP sensitisation appears to be different in melanoma and ovarian cancer cells.

Our data suggest that endogenous eNOS/nNOS activity in ovarian cancer cells, especially CDDP-resistant cells, produces low-level NO that protects them against induction of apoptosis, thus contributing to resistance against CDDP. Although iNOS could potentially also produce low-level NO favouring cytoprotection, the role of iNOS in ovarian cancer cells appears to be different from that of eNOS/nNOS; that is, iNOS contributes to, rather than suppresses, CDDP-induced apoptosis. This difference may relate to different localisation of the NOSs or different products generated by the NOSs (e.g., eNOS/nNOS generating cytoprotective NO, whereas iNOS generating cytotoxic forms of NO, such as peroxynitrite). Further studies will be needed to fully elucidate the different roles of the three NOSs in chemosensitive and chemoresistant ovarian cancer cells.

Overall, our data suggest a novel role for different NOSs in the regulation of apoptosis and sensitivity to CDDP in human ovarian cancer cells. The three NOS isoforms are regulated differentially by CDDP in CDDP-resistant and CDDP-sensitive ovarian cancer cells, which may contribute to chemoresistance. Inhibition of all NOSs in CDDP-resistant cells dramatically sensitises these cells to CDDP-induced apoptosis. The data suggest potential new ways of sensitising chemoresistant ovarian cancer cells to the therapeutic effects of CDDP.
